# Targeting Nurr1 With Amodiaquine Preserves Dendritic Spines and Cognitive Function After Chronic Cerebral Hypoperfusion

**DOI:** 10.1155/np/9997801

**Published:** 2026-05-25

**Authors:** Xiuli Zeng, Xiaomei Xie, Junrun Zhang, Jinyu Jia, Li’an Huang

**Affiliations:** ^1^ Department of Neurology, The First Affiliated Hospital, Jinan University, Guangzhou, China, jnu.edu.cn

**Keywords:** amodiaquine, chronic cerebral hypoperfusion, MRI, neuronal dendritic spine, Nurr1

## Abstract

**Background:**

Nurr1, an orphan nuclear receptor that lacks an endogenous ligand, plays a key role in hippocampal function, synaptic plasticity, and cognitive processes. It is linked to various central nervous system diseases; however, its association with chronic cerebral hypoperfusion (CCH)‐induced cognitive impairment remains unclear. This study investigated whether the Nurr1 agonist, amodiaquine (AQ), can enhance synaptic plasticity and alleviate cognitive deficits caused by CCH.

**Methods:**

A CCH rat model was created using the bilateral common carotid arterial occlusion method, followed by a 2‐week AQ treatment (20 mg/kg, every 12 h, through intraperitoneal injection). Learning and spatial memory were assessed using the Morris water maze (MWM), Y‐maze, and object recognition tests. Cerebral blood flow (CBF) in the cortex and hippocampus was measured using arterial spin labeling (ASL) with 3.0T magnetic resonance imaging (MRI), and white matter fiber density was assessed using diffusion tensor imaging (DTI). Hippocampal neuron morphology and count were examined using Nissl and NeuN staining, while dendritic spine morphology and density in CA1 and CA3 hippocampal regions were analyzed using Golgi staining.

**Results:**

Experiments with the water maze, Y‐maze, and object recognition tests demonstrated that CCH rats treated with AQ exhibited improved long‐term and short‐term memory and spatial recognition compared to the control group, with benefits persisting after two and 6 weeks. MRI DTI sequences revealed that AQ reversed the decline in white matter fiber density observed in CCH model rats compared to the sham group. Golgi staining confirmed that AQ protected the dendritic spines of the neurons damaged in the CCH model.

**Conclusion:**

Administrating Nurr1 agonist AQ demonstrated a sustained ameliorative effect on cognitive deficits in CCH rats. This effect is potentially mediated through the mitigation of hippocampal neuronal loss and improvement of dendritic spine integrity.

## 1. Introduction

The rat model of permanent bilateral common carotid artery occlusion (BCCAO) is frequently used in studies of chronic cerebral hypoperfusion (CCH) [1–3]. Our previous research indicated a significant decrease in cerebral blood flow (CBF) following BCCAO; however, cognitive deficits associated with CCH did not improve concomitantly with the restoration of blood flow [1]. This observation suggests the possibility of enduring damage to additional cerebral structures in CCH, which could represent a promising target for interventions aimed at ameliorating cognitive impairments.

Nurr1, a member of the orphan nuclear receptor family, is expressed in neurons across multiple regions of the central nervous system [4] and has been found to be responsive to pharmacological activation by small molecules [5]. Recently, significant focus has been placed on the role of Nurr1 in hippocampal synaptic plasticity, learning, and memory, prompting investigations into its potential involvement in brain pathologies associated with cognitive and intellectual impairment. Previous studies have demonstrated that hippocampal Nurr1 expression is elevated following memory‐inducing training [6, 7]. Additionally, Nurr1 overexpression has been found to ameliorate object location memory deficits in aged mice [8] and modulate the expression of genes associated with hippocampal synaptic plasticity and memory consolidation [9]. These findings imply that Nurr1 may have a significant impact on cognitive function, which is dependent on the hippocampus, although the precise mechanism remains unknown.

Our previous study established that Nurr1 messenger RNA (mRNA) expression is decreased in the cortex of rats with CCH, as determined by RNA sequencing [10]. Functional analysis revealed a significant correlation between the down‐regulation of Nurr1 mRNA and neurons and dendrites. Additionally, the absence of Nurr1 can result in deficits in hippocampal learning [11, 12] and memory and plays a role in the pathogenesis and cognitive function associated with Alzheimer’s disease [13]. However, the specific role of Nurr1 in CCH remains unclear. Hence, the present study used amodiaquine (AQ), a pharmacological agonist of Nurr1, to intervene in rats with CCH to investigate the impact and potential mechanism of AQ on CCH‐induced cognitive impairment.

## 2. Materials and Methods

### 2.1. BCCAO Surgical Procedure

The BCCAO model, a classic and well‐established model, was developed in accordance with previously outlined methodologies [1, 14] to study the molecular mechanisms and interventions of CCH. Briefly, rats were anesthetized with sodium pentobarbital administered intraperitoneally at a dose of 50 mg/kg. By entering the neck through a median incision, the skin, muscles, and fascia of the neck were separated layer‐by‐layer, followed by separation of the carotid sheath and vagus nerve and ligation with 4‐0 silk. The procedures for sham‐operated animals were identical, except for the absence of arterial ligation. A preheated cage was used to resuscitate the rats. Within ~3 h, the rats fully recovered and were placed back in a clean cage with full access to food and water.

### 2.2. Animals and AQ Treatment

Specific pathogen‐free Sprague‐Dawley (SD) rats (male, 250–300 g, 8 weeks old) were obtained from the Experimental Animal Center of Southern Medical University. During the experiment, the rats were housed at a temperature of 24 ± 2°C and provided free access to food and water with a 12/12‐h light/dark cycle. The experimental protocols were approved by the Laboratory Animal Ethics Committee of Jinan University and conducted in accordance with ethical standards (Approval Number IACUC‐20231201‐03). SD rats were intraperitoneally injected with AQ (Aladdin, A135245, 20 mg/kg) after BCCAO surgery, twice daily at 12 h intervals, for 2 weeks [15]. The first injection was administered on the first day after surgery, and the control animals were administered vehicle administration. The administration groups were labeled AQ2W (2 weeks of administration), AQ4W (2 weeks of administration followed by 2 weeks of natural elution), and AQ8W (2 weeks of administration followed by 6 weeks of natural elution). The control group’s reagents were named Vehicle2W, Vehicle4W, and Vehicle8W, respectively.

### 2.3. Morris Water Maze (MWM) Test

**Figure 1:**
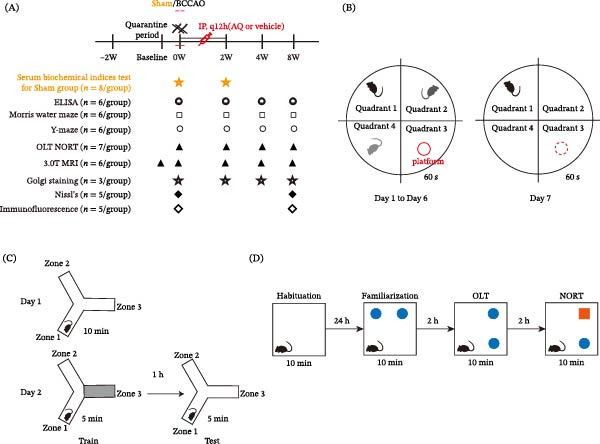
Overview of the experimental design process and behavioral experiment diagrams. (A) The experimental design process outlining the detection time points for various assessments. (B) Schematic representation of the Morris Water Maze apparatus. (C) Schematic illustration of the Y‐maze experimental apparatus. (D) Diagrammatic representation of the experimental design for novel location and object recognition assessments. BCCAO, bilateral common carotid artery occlusion; ELISA, enzyme‐linked immunosorbent assay; MRI, magnetic resonance imaging; NORT, new object recognition test; OLT, object location test.

The MWM test was used to evaluate long‐term spatial memory and learning in the rats (Figure 1B). The MWM test was conducted in a black circular pool with a diameter of 160 cm partially filled with water at a temperature of 23 ± 1°C. Experiment 1 involved positioning rats for training. For six consecutive days, the rats were trained in the same position three times in the first, second, and fourth quadrants, with their backs to the pool every day. Space exploration experiments were conducted on day 7. After removing the platform, the rats were released into the quadrant opposite the target quadrant (first quadrant). The rats were instructed to locate the platform within a 60‐s time frame, and their exploration path, frequency of crossings over the original platform location, and crossings within the quadrant containing the platform were documented. Rats that failed to swim or remained immobile were excluded from the MWM test.

### 2.4. Y‐Maze Test

The Y‐maze test was used to study the working memory of rodents in dynamic spaces, utilizing their natural exploration behavior in new environments (Figure 1C). The Y‐maze had three arms of equal size (50 cm × 18 cm × 35 cm) at a 120° angle with a movable partition in the center. The inner and bottom arms were black. After each session, the maze was cleaned with 75% alcohol to remove the rat odor. The experiment was conducted in a quiet, well‐insulated room under consistent light, temperature, and humidity conditions.

The experiment began with the spontaneous alternation behavior test, in which rats were given 10 min to freely explore a maze with three arms. The recorded metrics include the total number of entries, alternations, and maximum alternations. Percentage of spontaneous alternation = total alternation/maximum alternation × 100. The next test involved observing how the rats explored a new arm in a Y‐maze with different shapes as visual cues. The spatial recognition test was to record the exploration behavior of the new arm and evaluate the recognition ability of the new arm by closing one arm as a new arm. The Y‐maze was preset with three arms: Starting arm (Zone 1), the new alien arm (Zone 3), and the other arm (Zone 2). Blocking Zone 3 with a baffle allowed the rats to explore the other two arms. After 1 h, all arms were opened, and the rats were allowed to freely shuttle between them for 5 min. The time spent in the new arm and exploration time in the other arms were recorded and analyzed.

### 2.5. Object Location and New Object Recognition Test (NORT)

Rodents’ preference for novelty was used to assess their learning and memory abilities (Figure 1D). The experiments were conducted in a quiet environment with consistent light and humidity levels. A square behavioral box was used with objects placed 20 cm from the arm of the box. Three objects (A, B, and C) were prepared, with A and B being identical. The experiment had four phases, with the first being an adaptation period in which the test rats spent 10 min in a behavioral box before the experiment. In the familiarity stage, the rats were placed in a box with two identical objects and allowed to explore freely for 10 min. Contact times with the objects were recorded. In the third stage, the object location test (OLT) was conducted at 1 h intervals. Object B was moved to a diagonal position, whereas object A remained unchanged. The rats were allowed to explore freely for 10 min during their interactions with the recorded objects. The rats were then returned to their respective cages. The fourth stage was the NORT, conducted 1 h after training. Object A was replaced by object C, but its position remained the same. Rats were allowed to freely explore for 10 min, contact with the objects was recorded, and they were then returned to their cages.

Exploration of objects is defined as being within 2–3 cm of the object and includes activities such as sniffing, licking, and touching the front paws. Simply posing or climbing an object without interacting with it is not considered an exploration task. The recognition index (RI) was calculated as the evaluation criterion, with a new location RI = *T*_new_/(*T*_new_ + *T*_old_) and new object RI = *T*_novel_/(*T*_novel_ + *T*_familiar_), where *T* is the time spent exploring.

### 2.6. Golgi‐Cox Staining

The FD Rapid Golgi Stain Kit (FD Neurotechnologies, Inc.) was used to evaluate subtle morphological alterations in neuronal dendrites and dendritic spines. Specifically, the rat brain tissue was fixed in paraformaldehyde for a duration exceeding 24 h. Afterward, the designated experimental regions were sectioned into 2–3 mm thick brain tissue blocks, fully immersed in Golgi Cox stain, and subjected to a 14‐day incubation period (with stain renewal every 48 h and subsequently every 3 days). Tissue blocks were extracted and rinsed in distilled water for 1 min, immersed in 75% glacial acetic acid for 36 h, and then rinsed in distilled water for 1 min. Tissue blocks were then sectioned into slices measuring 100 microns using an oscillating microtome and affixed onto gelatin‐coated slides. The slices were left to air‐dry overnight. The following day, the tissue sections underwent a series of washing and treatment steps, including immersion in distilled water, concentrated ammonia water, and glycerin‐gelatin sealing. Thereafter, a panoramic image of the brain tissue was captured using a digital biopsy scanner (Nikon DS‐U3) and analyzed using the ImageJ software (Fiji). Neurons were examined at each location using a 100‐fold oil lens, with a focus on intercepting 6–8 dendritic spines on the secondary branches of basal dendrites located near the cell body, measuring ~20–30 μm in length. The dendritic spines were quantified.

### 2.7. Nissl Staining

Paraffin‐embedded tissue sections were dewaxed using xylene, followed by rehydration with a series of alcohol solutions. The sections were then immersed in the Nissl staining solution (Servicebio) for 5 min, rinsed with water, and dehydrated in xylene for 10 min to achieve transparency. After mounting the cover glass, the sections were examined under a microscope, and images were captured for analysis using the ImageJ software.

### 2.8. Immunofluorescence Staining

Following dewaxing and rehydration of paraffin sections, heat‐induced antigen retrieval was performed using tris‐EDTA buffer for 20 min. The brain sections were then blocked with 5% bull serum albumin for 1 h and then incubated overnight at 4°C with a mixture of rabbit NeuN (1:200, ab177487, Abcam). The following day, after rinsing with PBS, the tissue sections were incubated with a fluorescent secondary antibody in a light‐protected environment for 1 h. Following the addition of 4,6‐diamidino‐2‐phenylindole (DAPI) 2 HCl and placement of a cover glass, the sections were examined and imaged using a confocal microscope (LSM880, Zeiss).

### 2.9. Enzyme‐Linked Immunosorbent Assay (ELISA)

Following anesthesia, the ribcage was incised to extract whole blood from the right ventricle. Blood samples were allowed to stand at room temperature for 30 min, followed by centrifugation at 3000 revolutions per minute at 4°C for 15 min to separate the supernatant. Quantification of TNF‐α and IL‐1β was carried out in accordance with the manufacturer’s guidelines, and the levels of inflammatory cytokines were determined based on a standard curve.

### 2.10. Magnetic Resonance Imaging (MRI)

**Figure 2:**
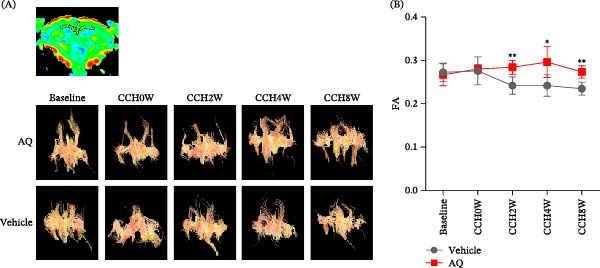
Investigation of the impact of amodiaquine on white matter injury in rats with CCH. (A) DTI sequences illustrating fiber bundle representations in the experimental groups following BCCAO. (B) Fractional anisotropy statistical maps derived from DTI sequences of the two groups post‐BCCAO. Sample size *n* = 6 per group. Statistical significance is indicated as follows:  ^∗^ *p* < 0.05, and  ^∗∗^ *p* < 0.01, comparing vehicle to AQ treatment. AQ, amodiaquine; CCH, chronic cerebral hypoperfusion; DTI, diffusion tensor imaging.

MRI with diffusion tensor imaging (DTI) was used to assess white matter integrity using a Discovery 750 3.0T scanner (GE Healthcare, Milwaukee, WI, United States). The groups treated with AQ and vehicle were subjected to MRI scans at six specific time points: Prior to occlusion, immediately after BCCAO, and at 2, 4, and 8 weeks post‐BCCAO (Figure 1A). The corpus callosum and both external capsules, which are regions abundant in the white matter tracts of the rat brain, were chosen for T2‐weighted imaging (Figure 2A). The 3D arterial spin labeling (ASL) technique was used to measure CBF in various brain regions of the rats (cortex and hippocampus). Detailed imaging parameters for the 3D ASL series, scanning procedures followed a previously established protocol, and data acquisition was conducted using previously described parameters [1, 2].

### 2.11. Statistical Analysis

Data processing and analyses were conducted using the Statistical Package for the Social Sciences software (Version 27.0). Fiji software was used to calculate the protein grayscale values and neuronal dendrite spine density. The GraphPad Prism software (Version 8.0) was used to generate the statistical graphs necessary for this study. Repeated measurement data for MWM escape latency, CBF, and DTI fractional anisotropy (FA) values were analyzed using a two‐way analysis of variance (ANOVA) with repeated measures. The remaining data were analyzed using one‐way ANOVA, with post hoc comparisons conducted using the least significant difference test (in cases of homogenous group variances) or Tamhane’s T2 test (in cases of uneven group variances). Statistical significance was set at *p* < 0.05.

## 3. Results

### 3.1. AQ Enhanced Learning and Memory Impairment Induced by CCH

**Figure 3:**
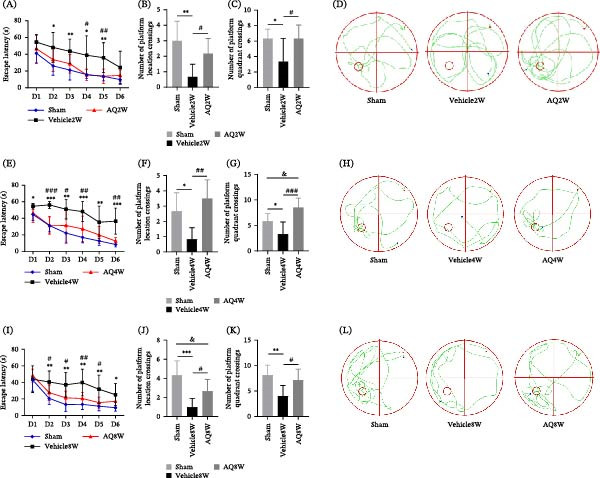
Effect of amodiaquine intervention on long‐term learning and memory capacity in rats with CCH. (A, E, and I) Escape latency observed in each rat group. (B, F, and J) Frequency of platform crossings in rats across trials. (C, G, and K) Frequency of quadrant crossings containing the platform for each rat group. (D, H, and L) Representative examples of rat swimming paths across the platform in each group. Sample size *n* = 6 per group. Statistical significance is denoted as follows:  ^∗^ *p* < 0.05,  ^∗∗^ *p* < 0.01,  ^∗∗∗^ *p* < 0.001 for sham versus vehicle comparisons; #*p* < 0.05, ##*p* < 0.01, ###*p* < 0.001 for vehicle versus AQ comparisons; &*p* < 0.05 for sham versus AQ comparisons. AQ, amodiaquine; D, day.

The Y‐maze, OLT, MWM, and NORT tests were conducted to evaluate the effects of AQ on CCH‐induced cognitive impairment. The swimming trajectories of all groups in the MWM experiment are demonstrated in Figure 3D,H,L, respectively. These findings suggest that rats in the AQ2W group demonstrated a decreased escape latency compared to those in the Vehicle2W group over the initial 6 days of training, as illustrated in Figure 3A. During the spatial exploration test, rats in the AQ2W group outperformed those in the Vehicle2W group in terms of platform location crossing (Figure 3B) and platform quadrant crossing (Figure 3C).

**Figure 4:**
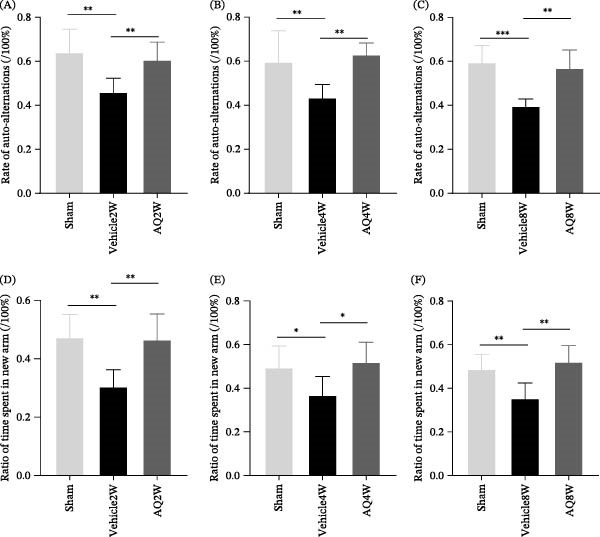
Effect of amodiaquine intervention on short‐term learning and memory capabilities in rats with CCH. (A, B, and C) Statistical analysis of Y‐maze spontaneous alternation rate in each group of rats. (D, E, and F) Statistical outcomes related to spatial recognition of the novel arms in the Y‐maze for each group. Sample size *n* = 6 per group. Statistical significance is denoted as follows:  ^∗^ *p* < 0.05,  ^∗∗^ *p* < 0.01, and  ^∗∗∗^ *p* < 0.001. AQ, amodiaquine; CCH, chronic cerebral hypoperfusion.

Rats in the AQ4W and AQ8W groups, which underwent drug‐eluting periods of 2 and 6 weeks, respectively, demonstrated significantly shorter escape times (Figure 3E,I) and superior performance in platform location crossings (Figure 3F and J) and platform quadrant crossings (Figure 3G,K) compared to the vehicle group during the same period. These results from the MWM experiment indicate that AQ has the potential to mitigate cognitive impairment in rats with CCH. The Y‐maze test was used to conduct a more comprehensive assessment of spatial learning and memory. The results revealed that treatment with AQ led to an increase in both the auto‐alternation ratio (Figure 4C) and the time spent in the new arm (Figure 4F) in rats with CCH, which persisted for up to 2 and 6 weeks after drug administration. The findings from the Y‐maze spontaneous alternation test and the new arm recognition test confirmed the presence of short‐term learning, memory, and spatial recognition deficits in rats with CCH. However, AQ intervention demonstrated a significant reversal in these short‐term cognitive impairments.

**Figure 5:**
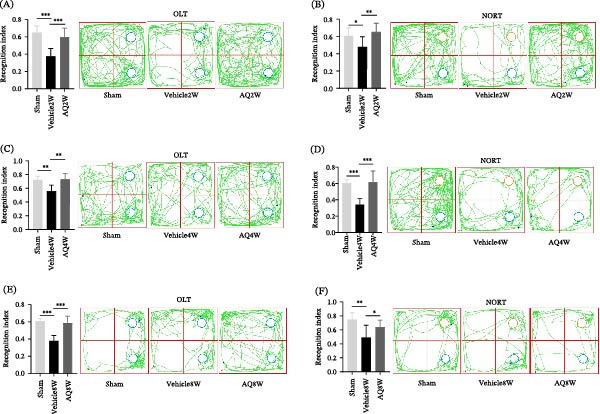
Investigation of the impact of amodiaquine intervention on novel location and object recognition in rats with CCH. (A, C, and E) Discrimination index and representative movement patterns for each rat group in the object location test. (B, D, and F) Discrimination index and representative movement patterns for each rat group in the new object recognition test. Sample size *n* = 7 per group. Statistical significance is indicated as follows:  ^∗^ *p* < 0.05,  ^∗∗^ *p* < 0.01, and  ^∗∗∗^ *p* < 0.001. CCH, chronic cerebral hypoperfusion; OLT, object location test; NORT, new object recognition test.

Rodents exhibit a natural inclination to explore novel stimuli, as evidenced by experiments involving unfamiliar environments and objects. Rats in the Vehicle2W group demonstrated decreased exploration of new stimuli compared to the sham group, suggesting a diminished capacity for recognizing novel stimuli in rats with CCH. Conversely, rats in the AQ2W group exhibited significantly prolonged exploration times for new stimuli, indicating superior recognition abilities compared to the Vehicle2W group (Figure 5A,B, *p* < 0.01), persisting for up to two and six weeks after drug administration. These results confirm that AQ improves recognition and sociability in rats with CCH.

### 3.2. AQ Improves CCH Cognitive Impairment by Repairing White Matter Damage, Not by Increasing Blood Flow

**Figure 6:**
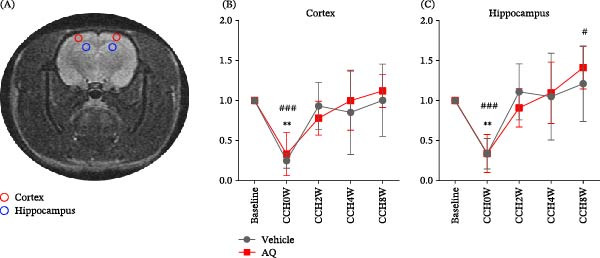
Effect of amodiaquine on CBF restoration in rats with CCH. (A) The designated regions of interest are illustrated, with the hippocampus highlighted in blue and the cortex highlighted in red. (B) Temporal changes in the cortical CBF. (C) Temporal changes in hippocampal CBF are presented. Sample size, *n* = 6 per group. Statistical significance is denoted as follows:  ^∗∗^ *p* < 0.01 for sham versus vehicle; #*p* < 0.05 and ###*p* < 0.001 for sham versus AQ. AQ, amodiaquine; CCH, chronic cerebral hypoperfusion.

This study used MRI observations to evaluate whether AQ could improve cognitive impairment in rats with CCH by enhancing CBF recovery. CBF in the cortex and hippocampus significantly decreased following CCH modeling (Figure 6B,C) and gradually recovered after 2 weeks. However, AQ intervention did not accelerate CBF recovery compared to the vehicle group. Although the AQ4W and AQ8W groups demonstrated a slight increase in CBF compared to the Vehicle4W and Vehicle8W groups, the differences were statistically nonsignificant.

DTI was used to monitor the white matter fibers, with the FA value closely linked to the integrity of the myelin sheath, fiber density, and parallelism. After BCCAO, a significant decrease in white matter fiber density was observed in the vehicle group compared to both the baseline and immediate postmodeling conditions (Figure 2B). Conversely, rats receiving AQ intervention displayed significantly preserved white matter fiber integrity compared to the vehicle group throughout the study period. This indicates that AQ may provide lasting protection against white matter damage caused by CCH, further highlighting its potential to alleviate cognitive impairment in rats with CCH.

### 3.3. AQ Intervention Significantly Alleviated Neuronal Dendritic Spine Injury in the Hippocampus of Rats With CCH

The necessity of synaptic plasticity in hippocampal neurons for learning and memory has been well‐documented. To investigate the potential of AQ to ameliorate cognitive deficits in rats with CCH by protecting neuronal dendritic spines from damage, we analyzed the density of neuronal dendritic spines in CA1 and CA3 regions of the hippocampus using Golgi staining.

**Figure 7:**
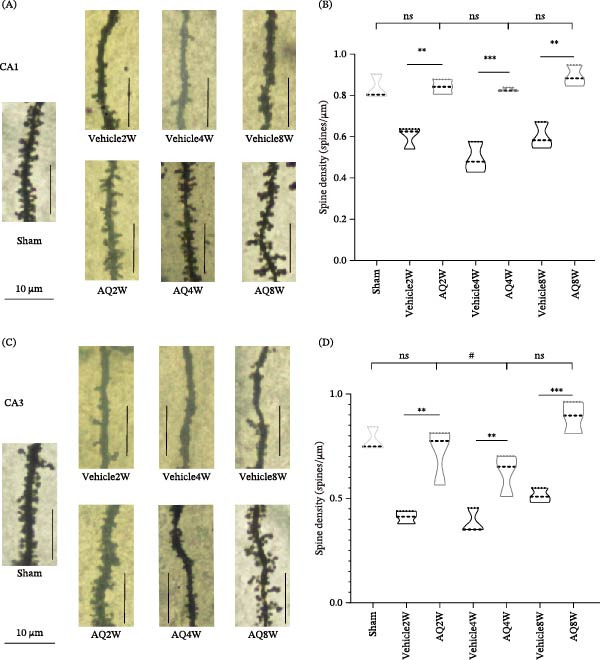
Impact of amodiaquine intervention on neuronal dendritic spines in the CA1 and CA3 regions of the hippocampus. (A) Representative images of neuronal dendritic spines in the CA1 region of rats from different experimental groups. (B) Quantitative analysis of the dendritic spine density in the CA1 region of each group. (C) Representative images of neuronal dendritic spines in the CA3 region of rats from different experimental groups. (D) Quantitative analysis of the dendritic spine density in the CA3 region of each group. Sample size *n* = 3 per group. Statistical significance is denoted as follows:  ^∗∗^ *p* < 0.01, and  ^∗∗∗^ *p* < 0.001 when comparing vehicle to sham; #*p* < 0.05 when comparing AQ to sham; ns indicates no significant difference **(** *p* > 0.05**).** AQ, amodiaquine.

The findings revealed a significant reduction in dendritic spines of neurons in the CA1 region of the hippocampus in the vehicle group compared to the sham group (Figure 7A), supporting the hypothesis that cognitive impairment in rats with CCH may be associated with a decrease in neuronal dendritic spines, as evidenced by morphological analysis. Additionally, treatment with AQ successfully alleviated neuronal dendritic spine damage in the CA1 region of rats with CCH (Figure 7B), as evidenced by comparisons with the vehicle groups (Vehicle2W, Vehicle4W, and Vehicle8W).

In the hippocampal CA3 region of rats, dendritic spines were mainly trunk‐shaped in the vehicle group, with reduced branching (Figure 7C). The AQ2W group exhibited a significant increase in dendritic spine density compared to the Vehicle2W group, with no significant difference compared to the sham group. Although the density of neuronal dendritic spines in the CA3 region was lower in the AQ4W group compared to the sham group, it was higher than that in the Vehicle4W group. The protective effect of AQ intervention on dendritic spines was more pronounced in the AQ8W group compared to the Vehicle8W group (Figure 7D).

### 3.4. AQ Intervention Downregulated the Expression of Inflammatory Factors in the Serum of Rats With CCH

**Figure 8:**
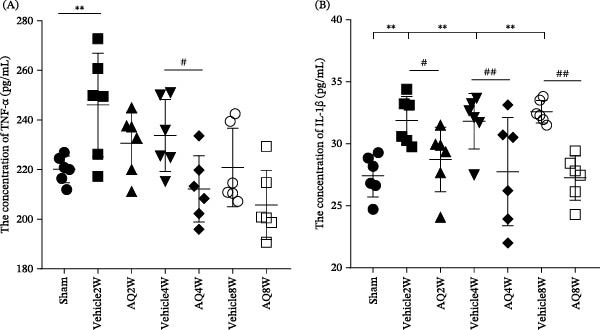
Investigation of the effect of amodiaquine intervention on serum inflammatory factor levels. (A) Serum TNF‐α levels were quantified by ELISA. (B) Serum IL‐1β levels were quantified using ELISA. Sample size, *n* = 6 per group. Statistical significance is indicated as follows:  ^∗^ *p* < 0.05, and  ^∗∗^ *p* < 0.01, relative to the sham group; #*p* < 0.05, ##*p* < 0.01, relative to the vehicle group. AQ, amodiaquine.

Serum levels of TNF‐α and IL‐1β were measured using ELISA. A significant increase in serum TNF‐α levels was observed in the Vehicle2W group (Figure 8A, *p* < 0.01), whereas no significant differences were noted in the Vehicle4W and Vehicle8W groups compared to the sham group. In the AQ2W group, TNF‐α levels decreased slightly following a 2‐week intervention with AQ; however, this reduction was statistically nonsignificant (*P* = 0.071). Notably, serum TNF‐α levels in the AQ4W group were significantly lower than those in the Vehicle4W group (*p* < 0.05). Analysis of serum IL‐1β levels revealed a significant up‐regulation in the Vehicle2W group compared to the sham group, whereas AQ intervention led to a reduction in serum IL‐1β levels in rats with CCH (Figure 8B, *p* < 0.05). Furthermore, serum IL‐1β levels in AQ4W and AQ8W groups were significantly lower than those in the corresponding Vehicle4W and Vehicle8W groups.

**Figure 9:**
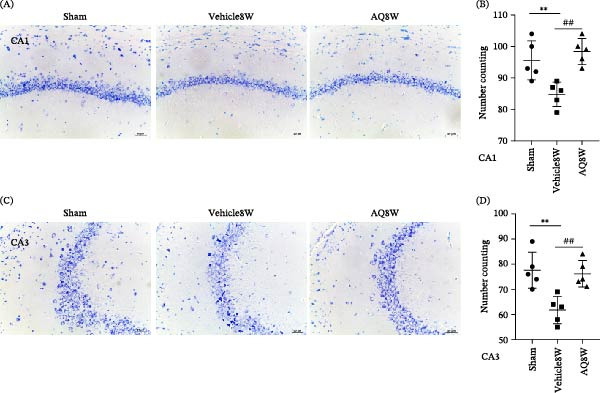
Effect of amodiaquine on Nissl staining‐positive hippocampal neurons in rats with CCH. (A) Representative Nissl‐stained images of the CA1 region. (B) Quantitative analysis of neuron counts in the CA1 region. (C) Representative Nissl‐stained images of the CA3 region. (D) Quantitative analysis of the neuronal counts in the CA3 region. Sample size, *n* = 5 per group. Statistical significance is denoted as follows:  ^∗∗^ *p* < 0.01, sham versus Vehicle8W; ##*p* < 0.01, vehicle8W versus AQ8W. AQ, amodiaquine.

Persistent cerebral hypoperfusion often leads to neuronal loss. Our findings demonstrated that AQ intervention consistently enhanced cognitive function in rats with CCH over 8 weeks. To explore the neuroprotective potential of AQ, hippocampal neurons were examined using Nissl staining and immunofluorescence at an 8‐week time point. The Vehicle8W group exhibited significant neuronal degeneration and reduction in the CA1 (Figure 9A) and CA3 (Figure 9C) regions of the hippocampus compared to the sham group. Conversely, AQ intervention significantly mitigated hippocampal neuronal loss in rats with CCH (Figure 9B,D; both *p* < 0.01).

**Figure 10:**
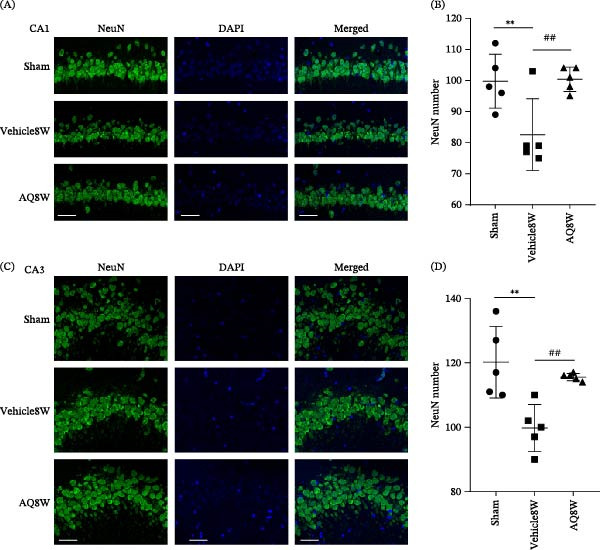
NeuN immunofluorescence staining and statistical analysis of hippocampal neurons in rats from each group. (A) Representative NeuN immunofluorescence staining in the CA1 region of the hippocampus, with NeuN and DAPI staining marked in green and blue, respectively. (B) Statistical analysis of NeuN‐positive cell counts in the CA1 region of the hippocampus for each group. (C) Representative NeuN immunofluorescence staining of the CA3 region. (D) Statistical map of NeuN‐positive cell counts in the CA3 region of the hippocampus of rats in each group. Sample size,*n* = 5 per group. Statistical significance is denoted as follows:  ^∗∗^ *p* < 0.01, sham versus Vehicle8W; ##*p* < 0.01, Vehicle8W versus AQ8W. AQ, amodiaquine.

To further demonstrate the impact of AQ on neuronal loss in the hippocampus of rats with CCH, as evidenced by Nissl staining, we conducted NeuN immunofluorescence labeling of CA1 and CA3 neurons. After 8 weeks of BCCAO, the Vehicle8W group demonstrated a significant reduction in NeuN‐positive cells in the CA1 and CA3 regions of the hippocampus compared to the sham group (Figure 10B, *p* < 0.01). Conversely, AQ intervention significantly increased the number of NeuN‐positive cells in the AQ8W group compared to the Vehicle8W group (Figure 10D, *p* < 0.01).

## 4. Discussions

Building on the findings of previous transcriptomic analyses [10], this study proposes that the pharmacological activation of Nurr1 can mitigate cognitive deficits caused by CCH through the alleviation of dendritic spine injury. The cognitive enhancement observed with AQ, a Nurr1 agonist, in the CCH model, was validated through a series of behavioral assessments (including MWM, Y‐maze, and new object recognition experiments), with the effects persisting for up to 6 weeks following the cessation of drug intervention. To investigate the underlying mechanisms, this study utilized DTI sequences and Golgi staining to demonstrate that long‐term cognitive improvements may be attributed to the preservation of white matter integrity and dendritic spine structure.

Nurr1 has demonstrated significant promise as a therapeutic target for Parkinson’s [5, 16, 17] and Alzheimer’s disease [13] as well as intracerebral hemorrhage [18]. AQ, a conventional antimalarial medication, has been identified as a Nurr1 agonist in numerous recent studies and is commonly utilized in various experimental models [5, 13, 16, 18]. In Parkinson’s disease models, AQ reduces neuroinflammation and enhances behavioral impairments, whereas in Alzheimer’s disease models [5], it decreases neuronal degeneration and amyloid‐beta accumulation [13]. Currently, there is limited research on the relationship between Nurr1 and CCH beyond the findings of our previous work [10]. Therefore, this study aimed to investigate the impact and mechanisms of Nurr1 agonists on cognitive deficits associated with CCH, using AQ as a pharmacological intervention.

The escape latency duration in the MWM experiment served as an indicator of learning capacity. Rats in the vehicle group exhibited longer escape latencies and fewer crossings in the platform location quadrant compared to the sham group, consistent with previous observations [1, 3, 19, 20], confirming cognitive deficits in rats with CCH. Conversely, the AQ group displayed a shorter latency in locating the hidden platform within 60 s than the vehicle group, suggesting that AQ mitigates the learning impairments induced by CCH. In the Y‐maze and novel object recognition tests, rats in the AQ group demonstrated improved rotation accuracy and enhanced recognition of new objects. Synaptic connections between neurons are dynamic and play a critical role in the expression and storage of memory [21]. These findings indicate that AQ intervention improves cognitive function in rats with CCH, likely through mechanisms such as synaptic pruning.

DTI is a cutting‐edge MRI technique that is highly sensitive to the directional diffusivity of water molecules, reflecting the orientation of white matter tracts [22]. In the rat brain, white matter tracts are most abundant in the corpus callosum and lateral exocallus. A reduction in FA values in these regions is indicative of axonal damage, as observed in the CCH model. Using DTI, our study confirmed a decrease in white matter density in rats with CCH, which is consistent with previous findings [10]. Following a 2‐week AQ intervention, a significant amelioration of white matter injury was observed. Additionally, mitigating astrogliosis caused by vascular stenosis in the CCH model helped preserve white matter integrity, thereby enhancing cognitive function [23]. Considering the integrity of white matter, it is plausible that other mechanisms, such as neuronal death, may also contribute to its degradation of white matter. In our study, AQ treatment significantly reduced neuronal loss in the hippocampus of rats with CCH.

Dendritic spinous structures undergo dynamic changes in response to normal conditions and synaptic plasticity and play a critical role in learning and memory [24]. Structural plasticity in the hippocampus facilitates the rebuilding of neural circuits [25]., which are essential for cognitive processes [25]. However, excessive elimination of dendritic spines can lead to a reduction in spine density, resulting in deficits in hippocampal‐dependent memory [26]. Conversely, an increase in dendritic spine density can enhance memory and neuronal plasticity [27]. AQ administration ameliorates lipopolysaccharide‐induced neuronal dendritic spine loss [28]. Previous studies on CCH have consistently reported varying degrees of neuronal dendritic spine damage in CCH‐induced rats [14, 29–31]. In this study, treatment with AQ effectively reversed the decline in dendritic spine density of multisite neurons in rats with CCH, and this effect persisted for up to 6 weeks after the cessation of treatment. These findings suggest that AQ can alleviate impairments in hippocampal neuronal structural plasticity caused by CCH. Moreover, previous studies have demonstrated that Nurr1 possesses anti‐inflammatory and neuroprotective properties [32, 33]. Consistent with these findings, our study observed that AQ administration significantly reduced the elevated levels of inflammatory factors in the serum of rats with CCH.

In this study, we provided novel insights into the effects of AQ in a CCH model through a combination of behavioral tests, MRI DWI, and Golgi staining. Our findings suggest that the sustained therapeutic effects of AQ in the CCH model may be attributed to the preservation of white matter integrity and dendritic spine structure. However, further cytological and mechanistic investigations were beyond the scope of this study. Future research should focus on designing Nurr1 probes or constructing Nurr1 gene model mice to demonstrate the regulatory role of Nurr1 in CCH.

The limitations of this study are as follows. First, no in vitro experiments were conducted to investigate the role and mechanism of Nurr1 in neuronal synaptic protection and neuroinflammation. Second, the mechanistic basis by which AQ upregulates Nurr1 in vivo to modulate synapse‐related pathways, thereby reducing neuronal synaptic damage and synergistically suppressing pro‐inflammatory cytokines such as TNF‐α and IL‐1β to improve cognitive function in CCH rats, was not explored in depth. Furthermore, the relationship between white matter damage assessed by MRI DTI, serum inflammatory factor levels, and cognitive impairment has yet to be validated in a clinical setting in patients with CCH; further studies are needed to elucidate this specific connection.

## 5. Conclusion

In summary, the Nurr1 agonist AQ demonstrated neuroprotective effects in rats with CCH, improved cognitive function, reduced white matter damage, alleviated neuronal loss, and mitigated neuronal dendritic spine injury. These findings provide strong preclinical evidence for Nurr1‐targeted regulation of neuronal synaptic damage, highlighting the potential value of repositioning the classic antimalarial drug AQ (as a Nurr1 agonist) as a therapeutic agent for cognitive impairment induced by CCH. With its sustained efficacy and high potential for clinical translation, this offers a new approach for the clinical treatment of CCH.

## Author Contributions

Xiuli Zeng authored the article and participated in neuropathological experiments. Xiuli Zeng and Xiaomei Xie conducted the experiments, while Xiaomei Xie and Junrun Zhang were involved in animal behavior experiments. Junrun Zhang and Jinyu Jia contributed to imageological examinations. Xiuli Zeng and Li’an Huang were responsible for study design and manuscript revisions.

## Funding

The research was funded by grants from the Guangzhou Science and Technology Project (Grant 2024A04J4175 to Xiuli Zeng and Grant 2025A03J4328 to Li’an Huang), the National Natural Science Foundation of China (Grant 82201438 to Xiaomei Xie and Grant 82571470 to Li’an Huang), the Guangdong S&T Program (Grant 2023B0303040003 to Li’an Huang), and the Major National R&D Project (Grant 2024ZD0527904 to Li’an Huang).

## Disclosure

All authors contributed to the article and approved the final version for submission.

## Conflicts of Interest

The authors declare no conflicts of interest.

## Data Availability

The data that support the findings of this study are available from the corresponding author upon reasonable request.
